# Lactate metabolism and lactylation in cardiovascular disease: novel mechanisms and therapeutic targets

**DOI:** 10.3389/fcvm.2024.1489438

**Published:** 2024-11-27

**Authors:** Han Zhang, Jiulu Zhao, Jizhang Yu, Xi Zhang, Shuan Ran, Song Wang, Weicong Ye, Zilong Luo, Xiaohan Li, Yanglin Hao, Junjie Zong, Ran Li, Longyong Lai, Kexiao Zheng, Pinyan Huang, Cheng Zhou, Jie Wu, Yuan Li, Jiahong Xia

**Affiliations:** ^1^ Department of Cardiovascular Surgery, Tongji Medical College, Union Hospital, Huazhong University of Science and Technology, Wuhan, Hubei, China; ^2^ Center for Translational Medicine, Tongji Medical College, Union Hospital, Huazhong University of Science and Technology, Wuhan, Hubei, China; ^3^ Key Laboratory of Organ Transplantation, Ministry of Education, NHC Key Laboratory of Organ Transplantation, Key Laboratory of Organ Transplantation, Chinese Academy of Medical Sciences, Wuhan, Hubei, China

**Keywords:** lactate metabolism, lactylation, immune cell, cardiovascular disease, glycolysis

## Abstract

Cardiovascular disease (CVD) is responsible for approximately 30% of annual global mortality rates, yet existing treatments for this condition are considered less than ideal. Despite being previously overlooked, lactate, a byproduct of glycolysis, is now acknowledged for its crucial role in the cellular functions of the cardiovascular system. Recent studies have shown that lactate influences the proliferation, differentiation, and activation of immune cells through its modulation of post-translational protein modifications, thereby affecting the development and prognosis of cardiovascular disease. Consequently, there has been a notable increase in interest towards drug targets targeting lactylation in immune cells, prompting further exploration. In light of the swift advancements in this domain, this review article is dedicated to examining lactylation in cardiovascular disease and potential drug targets for regulating lactylation, with the aim of enhancing comprehension of this intricate field.

## 1 Introduction

The image of lactate as a “metabolic waste” has been completely changed by the recent increase in research and understanding of lactate (1). Lactate is a key mediator of energy conversion in physiological and pathological metabolism, as well as participating in intricate cellular signaling mechanisms. Moreover, the effects of lactate before and after cardiac surgery has been thoroughly investigated (2).

The heart is undeniably a vital organ in the human body. While it relies mainly on the oxidation of carbohydrate like fatty acids and glucose to sustain its energy needs, lactate becomes an indispensable energy source under certain conditions, such as hypoxia or strenuous physical activity (3, 4). Under these conditions, the heart converts lactate from the blood into pyruvate through the action of the lactate dehydrogenase (LDH). Pyruvate then enters the tricarboxylic acid cycle to produce adenosine triphosphate (ATP), providing the heart with energy to maintain its function in an oxygen-deficient environment (5). A metabolic state dependent on lactate for energy production may not be the most optimal, but it serves as a vital adaptation that allows the heart to continue work even in conditions of limited oxygen supply (6, 7). However, excessive accumulation of lactate, especially in pathological states such as myocardial ischemia or myocardial infarction (MI), can lead to decreased intracellular pH, which affects heart function and even contributes to post-ischemia reperfusion injury (8). Recent studies have found evidence that lactate is a cardiac signaling molecule, regulating cardiac cells contractility and involving in the adaptive response of the heart during complex stress conditions (9). In-depth studies have shown that lactate may also affect the growth and survival of cells in the human heart, revealing its potential role in cardiac physiology and pathological regulation (10). Additionally, lactate is converted to lactyl-CoA, which then provides a lactyl group to the lysine residue with the assistance of lactyltransferase catalysis (11).

In conclusion, the significance of lactate in the human body extends beyond its involvement in energy metabolism. The identification of its contributions to cardiac energy supply, stress adaptation, signaling and lactylation modification represents a significant advancement in the comprehension of lactate function, offering a crucial biological foundation for a more comprehensive examination of the mechanisms underlying cardiac diseases and the formulation of novel therapeutic approaches (12).

## 2 Lactate metabolism and lactylation modification

### 2.1 Lactate metabolism under physiological condition in the cardiovascular system

The heart is capable of utilizing carbohydrates as a source of energy, and the significance of lactate in cardiac function is increasingly acknowledged. Lactate entering cardiomyocytes is oxidized to pyruvate by lactate dehydrogenase (LDH). Pyruvate then enters the mitochondria and is further oxidized through the tricarboxylic acid cycle (TCA cycle) to produce ATP, which provides the necessary energy for myocardial contraction (13). This is especially critical when the heart's workload increases. The heart effectively removes lactate from the blood through oxidative metabolism, helping to maintain acid-base balance throughout the body (14). For example, the net uptake of lactate by cardiomyocytes significantly increases during atrial pacing compared to the uptake at rest (15). Over the course of the experiment, it was observed that the myocardium exhibited an increase in the release of lactate, particularly during atrial pacing. These findings suggest that the heart not only utilizes lactate as a source of energy, but also releases it, with the proportion of release becoming more pronounced as the workload on the heart increases (16).

The monocarboxylate transporter (MCT) family, located on the lipid bilayer of the membranes, is responsible for the exchange of lactate inside and outside the cell and within the cell (17). Thus, lactate is dependent on MCTs to function in tissues or cells. Due to its role as symporters of lactate and hydrogen ions, MCTs is able to help regulate intracellular pH levels, thus contributing to pH homeostasis within cells. Fourteen MCT isoforms have been identified (18). From a tissue-level perspective, MCT1 expression is highly prevalent, with presence in various tissues including the heart, skeletal muscle, gastrointestinal tract, kidneys and so on. The uptake of lactate by the heart is dominated by MCT1. The localization of MCT1 on the cell membrane is dependent on the single-spanning transmembrane glycoprotein CD147, which is recognized as the chaperone protein for MCT1 (18, 19). Compared with MCT1, MCT2, MCT3 and MCT4 are expressed in different tissues. MCT4 has a low affinity for lactate and mainly promotes the excretion of lactate from cells (20). Consequently, MCT4 can promote the elimination of lactate generated through glycolysis (21). From a cell-level perspective, lactate is transported in human induced pluripotent stem cell-derived vascular smooth muscle cells (hiPSC-vSMCs) by MCT1 and MCT4 (22). The expression of MCT4, a crucial lactate transporter in cardiomyocytes, is notably increased during myocardial injury (10). MCT1 is also a major channel for lactate entry into endothelial cells (ECs).

**Figure 1:**
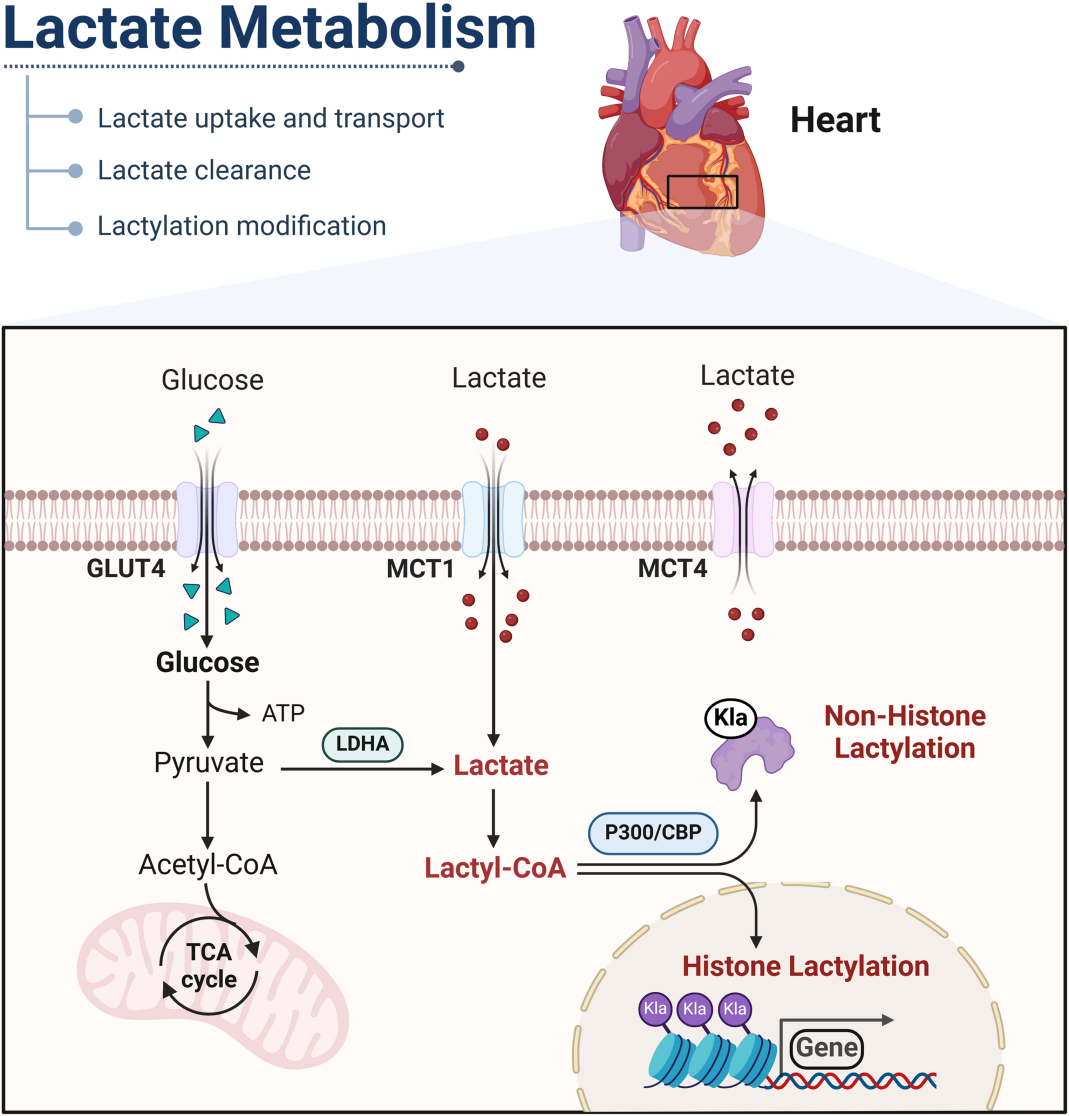
Mechanisms of lactate metabolism and lactylation in cells. Lactate is transported into the cytoplasm via MCT or produced through glycolysis. In the cytoplasm, lactate catabolism occurs through two pathways. In one pathway, lactate is oxidized to pyruvate, which enters mitochondria and is metabolized through the tricarboxylic acid cycle. In the other pathway, lactate can be converted into lactyl-CoA and is involved in the lactylation of histones and nonhistone proteins. GLUT, glucose transporter, Klalysine lactylation; MCT, monocarboxylic acid transporter; LDHA, lactate dehydrogenase A. This figure is created with BioRender.com.

In addition to glucose as the main energy supply, lactate can be converted into acetyl-CoA and subsequently enter the tricarboxylic acid cycle (TCA) to generate energy for the body. Furthermore, lactate has the capability to be converted into glucose through the gluconeogenesis pathway (23). It is important to recognize that the clearance of lactate by the kidneys plays a crucial role in maintaining the balance of NADH/NAD between cells and tissues. Lactate and pyruvate can undergo rapid interconversion facilitated by lactate dehydrogenase, enabling them to serve as circulating redox buffers. Furthermore, lactate can be converted into lactyl-CoA, which participates in the post-translational modifications of numerous proteins (24) (Figure 1).

### 2.2 Lactate metabolism under pathophysiological condition in the cardiovascular system

In pathological conditions, the balance between lactate production and clearance is often disturbed, leading to cellular dysfunction and potentially initiating a series of pathophysiological changes. This section will provide an overview of changes in lactate metabolism in various disease states. Specifically, in instances of acute myocardial infarction and congenital heart disease, there is a reduction in myocardial perfusion or arterial oxygen levels (25). The rate of glycolysis rises dramatically as the heart seeks to maintain ATP production. This leads to a rise in the production of lactate (26). However, the lack of blood flow prevents the removal of lactate from the cells (27). When lactate accumulates in the cells, it has a deleterious effect on ionic homeostasis and cardiac function, leading to disturbances in the contractile mechanism of the heart and cardiac electrophysiology (28, 29). During the development of atherosclerotic plaques, the arterial lumen gradually narrows, increasing the oxygen demand of the vessel wall, which in turn reduces the amount of oxygen diffusing to the intima. Due to hypoxia, intimal cells switch to glycolysis for energy production, resulting in increased lactate production (30). Currently, the reasons for elevated lactate levels in patients with hypertension are not clearly defined. It has only been found that lactate may promote the development of hypertension through increased central sympathetic nervous activity (31).

Hyperlactatemia is a prevalent issue following cardiac surgery and is often attributed to tissue hypoxia (type A). Type B (absence of tissue hypoxia) could also play a role after cardiac surgery (32). A normal blood lactate level is 0–2 mmol/L, and a value of 3–5 mmol/L is often used to define hyperlactatemia (33). And blood lactate levels and clearance rates have been found to be closely related to cardiac and vascular surgery prognosis. Patients with valvular heart disease in the intensive care unit, a serum lactate level >7 mmol/L and reduced lactate clearance are considered strong predictors of mortality within 30 days (34). Hyperlactatemia leads to the upregulation of vascular endothelial growth factor (VEGF), which can result in increased vascular permeability, tissue injury, hypotension, organ dysfunction and death (35). Hyperlactatemia is also associated with an increased risk of serious postoperative complications, including circulatory collapse and ventilation for >24 h, as well as a prolonged time to drain removal (36). Furthermore, studies have shown that the use of a cell saver during valve surgery can reduce blood loss and inflammatory responses. This significantly reduces the incidence of hyperlactatemia, which is beneficial for the treatment and long-term prognosis of surgical patients (37). Blood lactate levels feature prominently in acute type A aortic dissection (AAAD) patients (38). Surgery for AAAD is more complex and time-consuming compared to other cardiac surgeries. As a result, tissue ischemia and hypoxia are more severe, and lactate levels are higher (39, 40).

### 2.3 Lactylation modification

In addition to being a metabolite, lactate can also be used as a substrate for protein lactylation modification (11). Lactate is converted to lactyl-CoA, which then provides a lactyl group to the lysine residue with the assistance of lactyltransferase catalysis (41). In 2019, Zhang and colleagues identified lactylation of lysine residues (Kla) by analyzing core histones from human MCF-7 cells using high-performance liquid chromatography–tandem mass spectrometry. They identified 26 Kla sites in core histones from human HeLa cells and 16 Kla sites in histones from mouse bone marrow-derived macrophages (BMDMs) (42).

#### 2.3.1 Histone lactylation

Research has found that lactate, after being converted into lactyl-CoA, enters the nucleus and participates in the lactylation of histones, thereby regulating gene expression.

Elevated lactate levels have been shown to cause a notable rise in histone H3 lysine 18 lactylation (H3K18la) in the promoter area of the retinoic acid receptor γ gene, thereby inhibiting the transcription of this gene from chromatin. This downregulation was found to enhance TRAF6-IL-6-STAT3 signaling and reshape macrophage function (43). H3K18la promotes cancer cells proliferation and migration by regulating the expression of lipocalin-2 (44).

Histone H3 lysine 9 lactylation (H3K9la) is the major locus whose expression is upregulated in cells when lactate levels increase. Histone lactylation facilitates transcriptional activation primarily through modulation of the gene's transcription start site (45). The position of the sideroflexin 1 (SFXN1) promoter has been identified as an area of H3K9la enrichment. SFXN1 is a mitochondrial serine transporter required for one-carbon metabolism.

Subsequently, aberrant activation of SFXN1 promoted cells proliferation. Conversely, the suppression of SFXN1 arrested cell proliferation and halted the cell cycle (46). In addition, H3K9la may promote increased gene transcription in the PI3K-AKT pathway, the MPAK pathway, the Wnt pathway, and the Hippo pathway (45). H4K12la levels on the promoters of NF-*κ*B pathway genes, including *Ikbkb, Rela, and Relb*, are increased in animal models of ischemia-reperfusion injury. This leads to fibrosis of organs (47).

#### 2.3.2 Non-histone lactylation

The literatures have also been reported some sites which are not located on histone proteins, suggesting that lactylation is a universal modification not limited to histones and transcriptional regulation. Interestingly, the enzymes affected by lactylation are participated in metabolic processes, encompassing the TCA, as well as carbohydrate, amino acid, fatty acid, and nucleotide metabolism (48).

A specific instance is lactylation at the K28 site of adenylate kinase 2, which hinders its function and stimulates cell proliferation (48). Mitochondrial pyruvate carrier 1 (MPC1) affects the lactylation of fatty acid synthase (FASN) by regulating lactate levels in cells. Lactylation of the FASN K673 site inhibits FASN activity, which mediates the downregulation of lipid accumulation by MPC1 (49). Lactylation of insulin-like growth factor receptor-1 (IGF-1R) is reduced by the overexpression of Mucin 20 (MUC20) in cells. By reducing IGF-1R lactylation, MUC20 diminishes IGF-1R levels and its capacity to activate the receptor tyrosine kinase (MET) proto-oncogene, which is implicated in cell survival and proliferation (50). Lactylation of eEF1A2 at the K408 site can be catalyzed by KAT8 to enhance protein synthesis, which ultimately leads to the proliferation of cells. eEF1A2 is a protein essential for translation elongation (51). The latest research found that pyruvate kinase M2 (PKM2) is a substrate of lactylation modification. PKM2 is an irreplaceable molecule related to pro-inflammatory macrophage metabolism. Lactate enhances its pyruvate kinase activity by modulating the lactylation level of the K62 site on PKM2, leading to decreased glycolysis and suppression of the Warburg effect. This ultimately facilitates the shift of macrophages from a pro-inflammatory state to a reparative phenotype (52) (Table 1).

**Table 1:** Writers, erasers, readers, biological effects, and sites of novel PTMs.

Lactylation Enzymes	Lactylation site	Cell type	Related molecules or pathway	Downstream effect	Ref.
Writers					
P300/CBP	Pan-lactylation	Macrophages	ATF4/c-Jun pathway, ARG1	Polarization to M2 macrophages	(53, 54)
	H3K18	Th17 cells	FOXP3, IL-17	Promotion of reprogramming of Th17 cells	(55)
	Pan-lactylation	Epithelial Cells	TGF-β, Snail1	Promotion of endothelial-to-mesenchymal transition	(56)
	H3K18	Macrophages	Lrg1, Vegf-a and IL-10	Promotion of cardiac repair	(57)
	H3Kla	valve interstitial cells	Runx2, BMP2, TNF	Promotion aortic valve calcificaiton	(56)
KAT8	eEF1A2 K408	Cancer cells	eEF1A2	Increasement translational requirements for oncogenic adaptation	(51)
Not mentioned	Mecp2 lysine	Endothelial cells	Mecp2k271la, Ereg	Alleviates arteriosclerosis progression	(58)
Erasers					
HDAC1-3	Pan-lactylation	HeLa cells	Not mentioned	Cleave *ε*-N-L-lactyllysine marks	(59)
SIRT1	*α*-MHC K1897	myocardial cells	α-MHC and titin	Impairment of sarcomere stability	(10)

ATF4, activating transcription factor 4; ARG1, arginase 1; BMP, bone morphogenetic protein; Ereg, epiregulin; FOXP3, forkhead box protein P3; HDACs, histone deacetylases; IL-17, interleukin-17; KAT8, lysine Acetyltransferase 8; Runx2, runt-related transcription factor 2; TGF-β, transforming growth factor-β; TNF, tumor necrosis factor; α-MHC, α-myosin heavy chain.

#### 2.3.3 Writers, erasers and readers of lactylation

Like other epigenetic modifications, lactylation is regulated by specific enzymes known as “writers” (which attach lactyl groups to target proteins) and “erasers” (which remove these groups), working in conjunction with “readers” (proteins that recognize lactylation and assume related functions) (11).

The enzyme p300 has been implicated in histone lactylation (42, 60). When p300 levels decrease, there is a significant reduction in both lactate-induced histone lactylation and the activation of pro-fibrotic genes. This reduction was also observed specifically at gene promoters, indicating that p300 is crucial for enabling lactate to promote the expression of profibrotic genes via histone lactylation at relevant promoters (61). Histone lysine deacetylase enzymes HDAC1–3 and sirtuin (SIRT)1–3 can cleave *ε*-N-L-lactyllysine marks, with HDAC1–3 showing robust activity toward not only histone K(L-la) but also K(D-la) and various short-chain acyl modifications. Additionally, SIRT1 is identified as the delactylase for α-MHC K1897, with its expression reducing the degree of α-MHC lactylation (62). The de-L-lactylase activity of HDAC1 and HDAC3 suggested that histones are both inserted and removed by regulatory enzymes rather than by spontaneous chemical reactions (59).

HBO1 acts as a lactyltransferase, facilitating gene transcription dependent on histone Kla. The three genes AQP1, LAMC2 and F10 are all regulated by HBO1 (45).

There is insufficient research on “readers” of lactylation. To gain a more comprehensive and thorough understanding of their internal mechanisms and effects, future research endeavors must be further deepened and expanded.

Notably, the lactylation process catalyzed by the alanyl-tRNA synthetases AARS1 and AARS2 (AARS1/2) does not require the involvement of lactyl-CoA, but instead uses lactate directly as the donor of the lactyl group (63, 64). AARS1/2 is an ATP-dependent lactyltransferase that performs lactylation modifications through two consecutive reaction steps, utilizing ATP as an energy source. First, L-lactate reacts with ATP to form an intermediate lactate-AMP. In the second step, AARS1/2 transfers the lactyl group from the lactate adenylyl intermediate directly to specific lysine residues on target proteins (63). For instance, AARS1/2 can directly lactylate the lysine residues 131 and 156 on the N-terminus of cyclic GMP-AMP synthase (cGAS). Following lactylation, cGAS loses its ability to recognize double-stranded DNA (65, 66). This discovery provides a new perspective on understanding lactylation modifications.

## 3 Role of lactate and lactylation in cells

### 3.1 Lactate and lactylation regulate non-immune cells

#### 3.1.1 Vascular smooth muscle cells

Even under conditions of fully oxygen levels, vascular smooth muscle cells (VSMCs) produce significant quantities of lactate (67). VSMCs may favor glycolysis as a primary source of energy production. Recent studies have shown that high glucose conditions activate the transition of VSMCs to a synthetic phenotype, a process mediated through the lactate receptor GPR81, highlighting the lactate/GPR81 axis as a pivotal element in controlling this transition. Specifically, under high glucose conditions, GPR81 modulates the expression of the transcriptional coactivator PGC-1α, MCTs, and the cell surface glycoprotein CD147 in VSMCs (68). The phenotypic modulation of hiPSC-vSMCs induced by lactate might also be mediated by genes regulated downstream of N-myc (69, 70).

#### 3.1.2 Endothelial cells

Lactate promotes the activation of nuclear factor kappa B (NF-κB), which relies on PHD2 inhibition and ROS production. Moreover, NF-κB regulates the expression of interleukin (IL-8), which promotes angiogenesis (71). Lactate has also been shown to enhance VEGF production in ECs, leading to the induction of ECs migration (72).

### 3.2 Lactate and lactylation regulate immune cells in the heart

#### 3.2.1 T cells

T cells, as key cells in adaptive immunity, are significantly affected by lactic acid or an acidic environment. Therefore, understanding how lactic acid regulates T cell function is crucial for developing new immunotherapy strategies.

High lactate levels prevent the upregulation of nuclear factor in activated T cells in both T cells and natural killer cells, leading to decreased production of interferon-γ (73). In addition, high lactate levels impede T-cell receptor-triggered phosphorylation of JNK, c-Jun, and p38. The selective targeting of signaling proteins involved in interferon-γ production (JNK/c-Jun, p38) highlights the opposing effects of high lactate levels on cytotoxic T-cell responses (74).

However, lactate itself is an energy source for biological activity. Lithium carbonate promotes the localization of MCT1 to the inner mitochondrial membrane, allowing intracytosolic lactic acid to enter the mitochondria to provide oxidation and energy. Moreover, it is also involved in lactate metabolism after entering the mitochondria by activating transcription factor EB (TFEB). As a result, TFEB binds to the LDHB promoter, leading to increased LDHB expression. Pyruvate subsequently enters the TCA, where it undergoes oxidation to generate more energy to revitalize T cells (75).

Lactate also impedes CD4^+^ T cell motility by disrupting glycolysis, which is triggered when the chemokine CXCL10 binds to the chemokine receptor CXCR3 (55). In CD4+ T helper cells, lactate triggers a shift towards the Th17 subset which makes substantial amounts of the proinflammatory cytokine IL-17, improving fatty acid synthesis (55). The accumulation of lactate in diseased tissues promotes increased expression of the *Slc5a12* transporter on CD4^+^ T cells, which mediates alterations in the migration and function of CD4^+^ T cells. In CD8^+^ T cells, lactate causes the loss of cytolytic function (55). Thus, lactate-triggered signaling is an inhibitor of immune effector function (76).

#### 3.2.2 Macrophages

Macrophages are susceptible to polarization and phenotypic changes when stimulated by external conditions (77). Histone lactylation is one of the driving forces behind the repolarization of macrophages. In the later stages of M1, histone lactylation is linked to M2 gene expression and the concomitant silencing of M1 genes (78). Increased lactate levels during M1 polarization in macrophages result from a metabolic transition from oxidative phosphorylation to glycolysis, a process known as metabolic reprogramming (42). This metabolic switch is essential for macrophages to adapt to different stimuli and perform their functions effectively (79). Consequently, elevated intracellular lactate levels trigger histone lactylation and promote expression of M2 gene, events consistent with M1→M2 repolarization (53, 54, 80). In addition, lactate can directly induce lactylation of the nuclear high mobility group box-1 (HMGB1) protein via a mechanism dependent on the transcriptional coactivator p300/CBP. This leads to the transfer of HMGB1 from the nucleus to the cytoplasm in macrophages (80).

#### 3.2.3 Dendritic cells

Dendritic cells (DCs), which are professional antigen-presenting cells in the human body, have the capability to activate naive T cells (81). Previous research has shown that dendritic cells play a role in multiple cardiovascular diseases, encompassing hypertension, myocarditis, atherosclerosis, heart failure, and cardiac ischemia/reperfusion (82). Lactate-induced activation of HIF-1α leads to the upregulation of NDUFA4L2 [NADH dehydrogenase (ubiquinone)-1α subcomplex 4-like 2], which serves to restrain proinflammatory responses driven by mtROS and XBP1 in DCs, ultimately inhibiting T cell differentiation (83).

#### 3.2.4 NK cells

**Figure 2:**
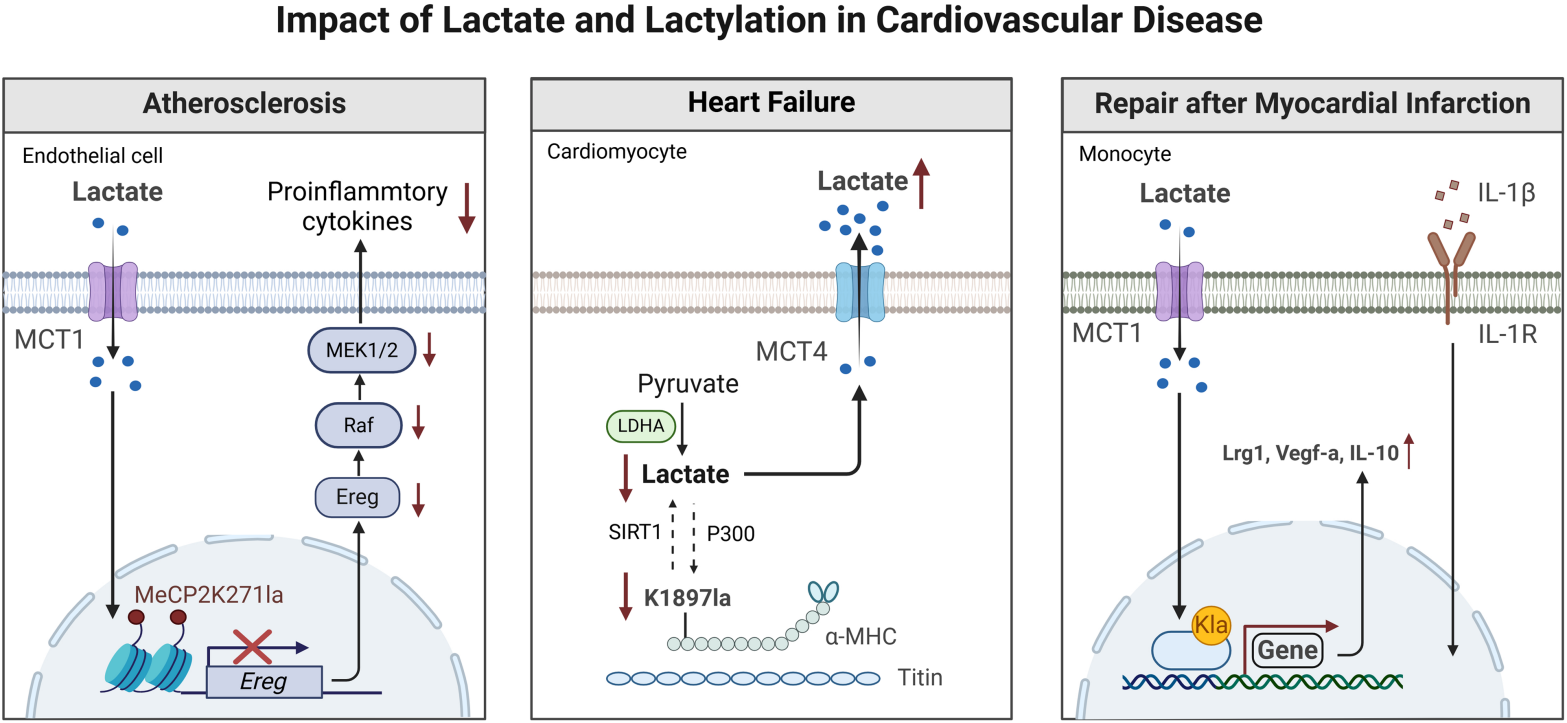
Effects of lactate and lactylation on the function of immune and non-immune cells in the cardiovascular system. GPR81, Gi-protein-coupled receptor 81; IL-8, interleukin-8; IL-10 interleukin-10; IL-17, interleukin-17; LDHA, lactate dehydrogenase A; M1/M2 type, M1/M2 macrophages; NF-κB, nuclear factor κB; VEGF, vascular endothelial growth factor; NAD^+^, nicotinamide adenine dinucleotide. This figure is created with BioRender.com.

Natural killer (NK) cells are involved in the development of cardiovascular diseases such as myocarditis, acute coronary syndrome, and cardiac fibrosis. In addition, in a mouse model of limited myocarditis, NK cells are vital in inhibiting viral infection and replication in the heart (84). Lactate can inhibit the upregulation of nuclear factor of activated T cells (NFAT). NFAT participates in the transcription of interferon-*γ*, thereby reducing the ability of NK cells to kill other cell (85). Lactate can also downregulate the NK cell surface marker NKp46, which in turn prevents the production of perforin/granzyme B, ultimately suppressing cellular cytotoxicity of NK cells. Lactate not only restricts the cellular cytotoxicity of NK cells but also hinders NK cells by boosting the numbers of myeloid-derived myeloid suppressor cells (86) (Figure 2).

## 4 Impact of lactate and lactylation in CVD

### 4.1 Heart failure

Heart failure (HF) is a complex, chronic CVD marked by diminished capacity of the heart to fill with blood and pump (87). Clinically, elevated blood lactate is common in patients with HF and is associated with clinical markers of organ dysfunction and a poor prognosis (40, 88). Lactate is capable of affecting every link in the excitation-contraction coupling from action potential generation to contraction produced by cross-bridge oscillations (89). However, some researchers have observed that within cardiomyocytes of heart failure patients, lactate levels demonstrate a completely opposite trend. This discovery has prompted them to question the mechanism by which lactate functions in heart failure (40). This reduction leads to less lactylation of α-myosin heavy chain (α-MHC) K1897 and a significant decrease in the interaction between α-MHC and the protein titin. Since the connection between α-MHC and titin is critical for preserving sarcomere stability, reduced interaction between the two ultimately leads to HF (90).

Notably, since the expression of MCT4 was significantly upregulated during myocardial injury, inhibiting this transporter could increase α-MHC K1897 lactylation, reduce myocardial fibrosis, and alleviate HF (10). Various MCT inhibitors have been shown to decrease intracellular lactate efflux, enhance *α*-MHC K1897 lactylation, and relieve HF, making them potential treatment options (62).

### 4.2 Atherosclerosis

Atherosclerosis is a risk factor for acute ACS, including MI. Current studies have shown that atherosclerosis is a chronic, immunoinflammatory, fibroproliferative disease (91). Vascular ECs, macrophages, and VSMCs are pivotal for disease progression. The role of lactate in ECs has been clarified. In mouse aortic ECs, Mecp2k271la was enriched in the epiregulin (Ereg) promoter region and inhibited the transcription of Eregs and the expression of Ereg mRNA (92). Research indicates that Ereg can promote the phosphorylation of epidermal growth factor receptor and activate the MAPK signaling pathway. Therefore, Mecp2k271la overexpression suppressed the activation of the MAPK signaling pathway induced by Ereg, leading to decreased expression of proinflammatory cytokines and decreasing the mRNA expression levels of proinflammatory cytokines such as IL-1β, IL-6, vascular cell adhesion molecule 1, intercellular adhesion molecule 1, and monocyte chemoattractant protein 1, as well as the levels of proinflammatory cytokines in the serum (93).

In addition, the increase in the adhesion of ECs to macrophages induced by Eleg can be inhibited by Mecp2k271la, reflecting the anti-inflammatory effect of lactate-induced Mecp2k271la on ECs. Most importantly, treatment with lactate substantially decreased plaque lesions in ApoE^−/−^ mice (58).

### 4.3 Myocardial infarction

The prognosis of patients with MI is strongly correlated with lactate levels, and hyperlactatemia increases mortality at 30 days postinfarction (94). Patients who died exhibited a markedly increased lactate/albumin (L/A) ratio in comparison to those who survived (95, 96). These findings suggest that the effect of lactate on heart function after MI is particularly important.

Recently, aerobic glycolysis has also been found to play a factor in ischemia‒reperfusion injury (97). Under hypoxia/reoxygenation (H/R) conditions, the reduction in extracellular lactate levels and the expression of glycolysis-related genes (*GLUT4* and *LDHA*) are exacerbated following the knockdown of heat shock protein A12A (*HSPA12A)* (98). HSPA12A maintains histone 3 lactylation by increasing the stability of the Smad-specific E3 ubiquitin ligase 1-mediated hypoxia-inducible factor 1α protein, which supports aerobic glycolytic homeostasis, thus maintaining the survival of cardiomyocytes under H/R stimulation (98).

**Figure 3:**
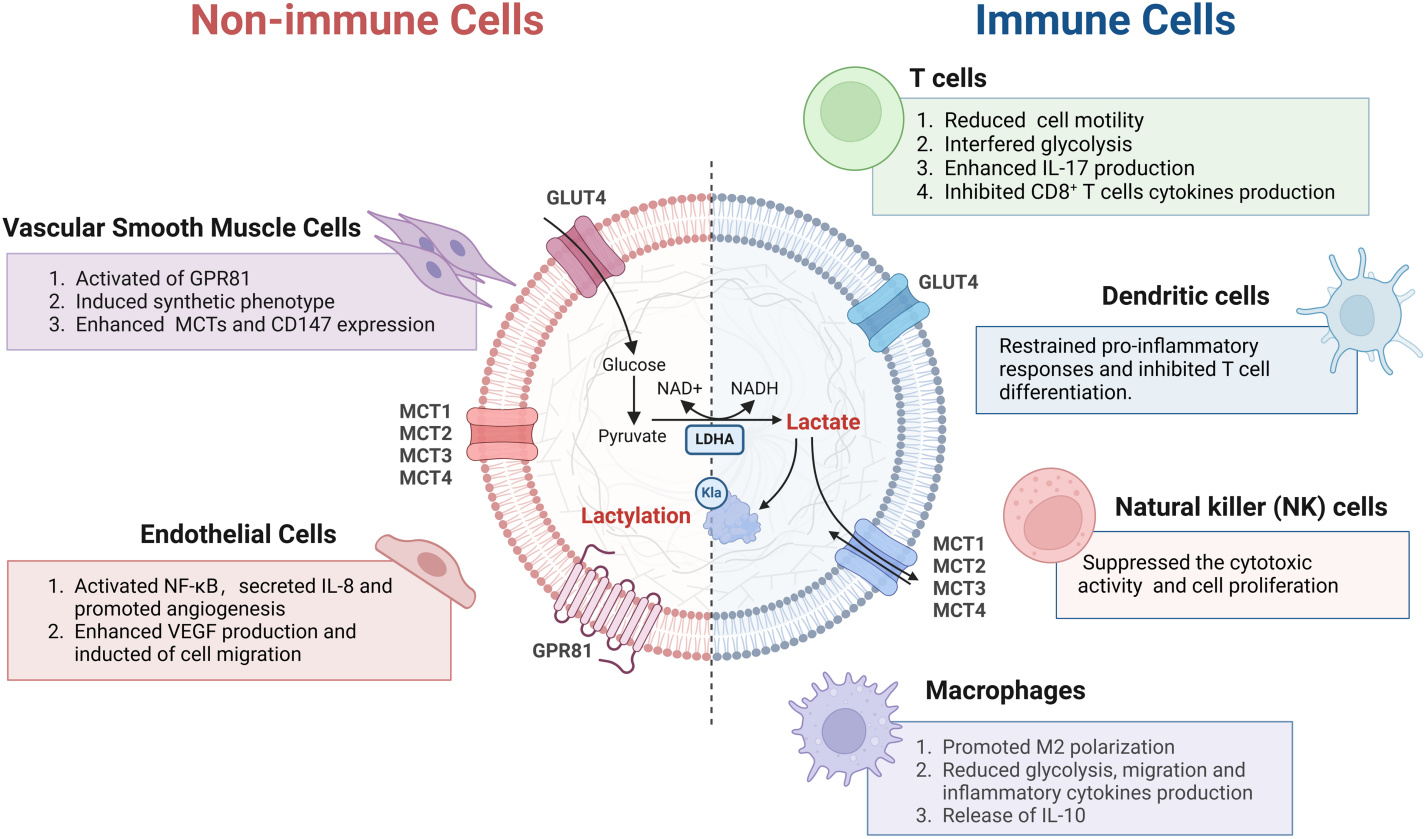
The role of lactate and lactylation in cardiovascular disease. **(A)** Lactate and lactylation maintain sarcomere function to alleviate the development of heart failure. **(B)** Lactate and lactylation suppress atherosclerosis. **(C)** Lactate and lactylation promote activation of the cardiac repair process post-myocardial infarction. α-MHC, α-myosin heavy chain; Ereg, epiregulin; IL-β, interleukin-β; SIRT1, sirtuin 1. This figure is created by BioRender.com.

After MI, monocytes have a high glycolytic metabolic capacity. They control histone lactylation through the provision of lactate as a substrate, resulting in enhanced expression of the lactylation-related modification and lactylation target genes *Lrg1*, *Vegf-a*, *and Il-10*. These genes have pro-angiogenic and anti-inflammatory effects (99). Moreover, increase in H3K18la inhibited the production of the inflammatory cytokines IL-6 and tumor necrosis factor, which led to a decrease in inflammatory cell infiltration, prevented excessive myocardial fibrosis and pathological cardiac remodeling, and improved cardiac function post-MI (99). However, another study showed that after hypoxia or MI, MCT-mediated extracellular lactate enters the cell, and lactate induces the lactylation of Snail1 (a transcription factor of transforming growth factor TGF-β) and nuclear chromosomal translocation (100). The binding of the Snail1 protein to the *TGFB1* gene activates endothelial-mesenchymal transition via the TGF-β/smad2 signaling pathway, resulting in myocardial fibrosis and the aggravation of cardiac dysfunction (101). In addition, lactate has been found to promote the synthetic phenotype of VSMCs, which increases lactate levels due to ischemia‒reperfusion injury, increases the expression of MCT1 and MCT4 after lactate increases, and promotes VSMCs proliferation. This mechanism may be utilized to improve myocardial repair in certain types of ischemic heart disease (57) (Figure 3).

### 4.4 Aging heart

As early as 2003, researchers found that plasma lactate levels in the elderly are significantly higher than those in younger individuals (102). This phenomenon exists in humans and can likewise be observed in aging mice. In the hearts of aging mice, notable metabolic remodeling occurs, characterized by a reduction or even inhibition of lactate oxidation. Furthermore, with age increases, the decline in lactate oxidation becomes more pronounced (103). Additionally, studies have identified a mitochondrial enzyme, cardiac succinyl-CoA-3-oxoacid CoA transferase (SCOT), which significantly elevates glucose and lactate levels in mouse plasma. The activity of this enzyme dramatically increases in aging mice (104). Changes in lactate metabolism are evident not only in its output but also in its transport processes. In the aging heart, cellular crosstalk between cardiac fibroblasts and cardiomyocytes inhibits the expression of lactate transporters, potentially linked to the fibroblast growth factor 21 (FGF21)-adiponectin pathway's role in the aging process (105, 106). This may occur through the regulation of a series of downstream signaling molecules, indirectly affecting the function of lactate transporters. Overall, alterations in cell-cell communication and signaling pathways may exacerbate lactate metabolism disorders, leading to lactate accumulation and subsequently impacting cardiac function (104).

### 4.5 Valvular heart disease

According to reports, valvular heart disease is one of the common diseases in cardiovascular surgery, accounting for about one-third of cardiovascular surgery patients in China (107). Researchers have concentrated on the role of lactylation modification in valvular heart disease and have elucidated several molecular mechanisms associated with disease progression (3). For example, Lumican, a component widely present in the extracellular matrix (ECM), regulates fibrosis and calcification in the heart and other tissues (108, 109). Numerous studies have shown that the occurrence and development of inflammatory diseases and atherosclerosis are linked to Lumican, suggesting that valvular heart disease may share a similar etiological factor (110, 111). In valvular heart disease, elevated levels of Lumican increase inflammatory pathway responses and enhances cellular glycolysis, leading to increased lactate production. Lactate, as a substrate, promotes histone H3 lactylation modifications, particularly at the H3K14la and H3K9la sites, driving the expression of calcification-related genes such as Runx2 and BMP2, which accelerates the progression of calcific aortic valve disease (CAVD). Lumican deficiency effectively inhibits the calcified deposits in the aortic valves and related symptoms (112). Wang et al. found that andrographolide (AGP) inhibits H3Kla, reduces the expression of Runx2 and BMP2 by interfering with the activity of p300, and diminishes calcified deposit in valvular interstitial cells (VICs), thus alleviating CAVD (56). Lumican and AGP provide a theoretical foundation for early pharmacological intervention in heart valve disease.

## 5 Targeting lactate metabolism or lactylation

### 5.1 Lactate dehydrogenase inhibitors

Lactate dehydrogenase A(LDHA) plays a crucial factor in the metabolic process of converting pyruvate into lactate during glycolysis and becomes a therapeutic target for a variety of diseases. Drugs that inhibit LDHA, such as galloflavin, vitamin C, and sodium oxamate, have been developed to interfere with lactate production (113, 114). In homogenates of pulmonary arteries from hypoxic rats, elevated LDH activity and lactate levels were decreased following treatment with oxamate. Increased levels of the histone lactylation marks H3K18la and H4K5la in these homogenates and in tissues isolated from hypoxic rats were also reduced by oxamate (114).

### 5.2 MCT inhibitors

MCTs inhibitors alter the intracellular metabolic state by disrupting the lactate shuttle. Extensive research is currently underway for the use of MCTs inhibitors in the cardiovascular system as well as in other systems (94, 95).

#### 5.2.1 α-Cyano-4-hydroxycinnamic acid

α-Cyano-4-hydroxycinnamic acid (CHC) is a classic, nonselective inhibitor of MCTs (115, 116). When CHC blocks lactate uptake by macrophages, it also suppresses lactate-induced increases in Klac in macrophages (117). Inhibition of MCT1 by CHC causes complete blockade of lactate uptake in cells, resulting in the cessation of lactate-fuelled respiration. Importantly, CHC does not affect cell respiration in the presence of glucose. These results suggest that CHC has an inhibitory effect on mitochondrial function when MCT1 is involved in lactate uptake for oxidative metabolism (118).

#### 5.2.2 AZD3965

MCT1 shows a higher affinity for lactate and can operate to either import or export lactate when expressed in oxidative or glycolytic cells, respectively (119). Therefore, MCT1 is an attractive drug target (118). AZD3965 inhibits MCT1 transporter activity by binding to the key MCT1 residues Lys38, Asp302, and Arg306, which are involved in lactate transport within transmembrane helices (120). The interaction between MCT1 and CD147/basigin is essential for the selectivity of AZD3965 towards MCT1, which aids in drug accessibility or binding (121).

#### 5.2.3 Syrosingopine

MCT4 facilitates the expulsion of both lactate and protons from the cell. Inhibiting MCT4 with syrosingopine is expected to result in intracellular lactate accumulation, accompanied by decreased glucose consumption, intracellular acidification, and reduced lactate secretion (54). The high concentration of lactate in cells prevents the continuous conversion of NADH into NAD+. The combined application of syrosingopine, metformin, and other drugs has demonstrated significant efficacy in treating a variety of diseases, indicating promising therapeutic potential (122).

### 5.3 Histone lactylation inhibitors

The transcription coactivator p300/CBP was reported to possess intrinsic histone lactyltransferase activity and to catalyze the transfer of the lactyl group from lactyl-CoA to histones in a cell-free system (42, 123). The compound C646 was shown to be a linear competitive inhibitor of p300 over acetyl-CoA, and the reaction process was reversible (124). Researchers used C646 to suppress the activity of p300/CBP and noted that this suppression significantly attenuated the lactate-induced increase in Klac levels in macrophages (80) (Table 2).

**Table 2:** Potential drugs that target lactate metabolism and lactylation.

Target	Representative drugs	Mechanism of action	Research status	Ref/Trial No.
Lactate metabolism intervention				
LDHA/LDHB	AT-101	Inhibits LDHA/LDHB		
LDHA	RS6212	Inhibits LDHA	Preclinical	(125)
	FX-11	Inhibits LDHA	Preclinical	(5)
	GSK2837808A	Inhibits LDHA	Preclinical	(126)
	Oxamate	Inhibits LDHA	Preclinical	(114)
Lactate transporters	a-Cyano-4-hydroxycinnamic acid	Inhibits MCT1/2	Preclinical	(115, 116)
	AZD396	Inhibits MCT1/2	Clinical trail	NCT01791595
	Syrosingopine	Inhibits MCT1/4	Preclinical	(122)
	VB124	Inhibits MCT4	Preclinical	(57)
	AZD1422	Inhibits MCT4	Preclinical	(127)
	7ACC2	Inhibits MCT4	Preclinical	(128)
CD147	Meplazumab	Interferes the distribution of MCT1/4 on cell membrane	Clinical trail	NCT06040346
Hexokinase	2-deoxy-D-glucose	Inhibits hexokinase	Clinical trial	NCT00096707, NCT00588185, etc.
	Lonidamine	Inhibits glycolysis	Preclinical	(129)
Lactylation modification intervention				
P300/CBP	C646	Inhibits lactylation production	Preclinical	(83, 121)
	A-485	Inhibits lactylation production	Preclinical	(123)
	I-CBP112	Inhibits lactylation production	Preclinical	(85, 130)
SIRT1–3	TSA	Promotes lactylation production	Clinical trial	NCT05563948, NCT05473429, etc.
HDAC1–3	nicotinamide	Promotes lactylation production	Clinical trial	NCT04614714

CBP, the CREB-binding protein; LDHA, lactate dehydrogenase A; HDAC, histone deacetylase; MCT, monocarboxylate transporter; SIRT1–3, sirtuin 1–3; TSA, tricho.

### 5.4 Limitations

The current treatment of hyperlactatemia primarily focuses on addressing the underlying causes, without considering the long-term implications of hyperlactatemia and lactylation modification on CVD. The timing and efficacy of treatment for severe hyperlactatemia remain contentious. Most studies indicate that when the pH drops below 7.1, it can lead to cellular metabolic dysfunction, inhibition of the cardiovascular system, and diminished responsiveness to catecholamines. Sodium bicarbonate should be administered promptly to mitigate metabolic acidosis and restore normal cellular function; for patients with pH levels between 7.1 and 7.2, particularly those with severe acute kidney injury, sodium bicarbonate is also warranted (131–134). However, other studies suggest that sodium bicarbonate does not enhance cardiovascular function or reduce mortality (135). While carbon dioxide can traverse the cell membrane, the movement of bicarbonate into cells is hindered, potentially inducing intracellular respiratory acidosis. This underscores the need for caution when administering sodium bicarbonate to patients with hyperlactatemia (11, 132). In addressing this issue, Jeffrey A. Kraut discovered that hyperventilation and calcium infusion can enhance the cardiovascular function of animals during sodium bicarbonate administration, suggesting that these methods may optimize the therapeutic effects of sodium bicarbonate. Additionally, other buffers (such as tris-hydroxymethyl aminomethane or Carbicarb) or dialysis may help mitigate adverse effects. These findings necessitate further validation through clinical trials (132).

## 6 Conclusions

In this review, we have highlighted the key findings and advancements in the field of lactate metabolism, lactylation and drug treatment targets in cardiovascular disease. Lactylation can affect the function of various cells and progression of CVD through multiple mechanisms, including but not limited to regulating cell metabolism, influencing inflammatory responses, and participating in the repair process. These findings offer new perspectives and targets for the treatment of CVD.

The current understanding of the mechanism of lactylation in MI, HF, atherosclerosis and valvular heart disease (such as aortic valve disease) remains in the early stages. The mechanisms by which lactylation modification regulates cellular metabolism in hypertension, aortic dissection and infective endocarditis remain largely unexplored and require further research. In particular, hypertension, one of the most prevalent cardiovascular diseases, necessitates urgent exploration of the mechanisms by which lactylation affects this condition. Furthermore, understanding the interplay between lactylation and other cellular processes could unveil multi-target strategies that address the complex nature of CVD. Such approaches promise to open new avenues for the treatment of cardiovascular diseases, potentially leading to more effective and personalized therapies. Numerous technologies are now available for the study of lactylation, including gene editing, CUT&Tag and bioinformatics, which are poised to become significant directions for future research.
